# Writing for SICOT-J

**DOI:** 10.1051/sicotj/2021042

**Published:** 2021-08-16

**Authors:** Andreas F. Mavrogenis, Isabelle Auffret Babak, Jacques H. Caton

**Affiliations:** 1 First Department of Orthopaedics, National and Kapodistrian University of Athens, School of Medicine 11527 Athens Greece; 2 EDP Sciences 91944 Les Ulis France; 3 Institut de Chirurgie Ortopédique 69727 Lyon France

**Keywords:** Medical writing, Instructions to authors, Outline, Authorship, Submission

## Abstract

Every time a paper is submitted to the journal, we realize the effort and amount of work it takes for performing the study, writing, formatting, and submitting the paper for peer review. However, how many of these papers are suitable for publication? Medical writing considerations, including an understandable text that does not confuse reading, formality, and ethics in writing, should be kept in mind when preparing and writing a paper to be submitted for publication to a journal. This editorial note offers useful advice for the authors submitting their papers to a journal on what to keep in mind before submission, how to prepare a quality submission, how to win the editor for their paper to avoid rejection, and how to make it to the review process and maybe to get published. It is our belief that these tips and advice on medical writing apply to any author and any journal.

## Introduction

Science progresses by the publication of novel ideas and research. It does not exist unless it is published. However, medical writing has special considerations; it should be formal, accurate and up-to-date, unbiased, easy to understand, ethical, should not confuse reading, and adhere to high-quality principles and guidelines. Every time a paper is submitted to a journal, the Editors realize the effort and amount of work it takes for performing the study, writing, formatting, and submitting the paper for publication. However, how many of these papers are accepted for publication? Medical writing has special considerations; it should be easy to understand, should not confuse reading, and should be ethical and formal in writing. These should be kept in mind when preparing and writing a paper to be submitted and considered for publication to a journal.

This article offers valuable advice for the authors submitting their papers to a journal on what to keep in mind before submission, how to prepare a quality submission, the prerequisites (checklist) for an attractive submission, how to win the editor for their paper to avoid rejection and to make it to the review process. These tips on medical writing apply to any author and any journal and focus scientists on submission and publication of quality papers.

## Outline

An outline is a short, organized description of what will be contained in an article. It is important to organize the work and make writing and reading simple. The classic hierarchal model of the outline is the IMRAD system (Introduction, Methods, Results, and Discussion) used for research reports [1]. The length and complexity of the outline depending on the length and complexity of the paper. For a short article, an extensive outline with multiple topics is unnecessary because it complicates and confuses reading. In contrast, a more extensive outline is necessary for a longer article to organize data in a structured manner and make reading simpler.

## Instructions to authors

The instructions to authors should be followed concerning the manuscript and the references’ format, and medical writing. The authors should not begin writing without reading the instructions to authors for the journal in question. Most instructions describe the types of articles published, provide guidelines for preparing each type of article, and list requirements for submitted papers. The authors should consult these instructions because failure to do so results in extra work for the editors and the authors and can cause delays in the review process. The manuscript is sent back to authors for the missing information or correction of the wrong format. Moreover, failure to follow instructions can result in rapid rejection, simply because by the authors carelessly prepared it.

Based on the journal’s instructions, requirements for manuscript submission may be related to ethical standards (IRB approval, Patients’ informed consent, Disclosures). The editors reserve the right to reject manuscripts that do not comply with these requirements. All medical writing contains references, and although each publication has its reference style, most follow the International Committee of Medical Journal Editors (ICMJE) [2]. The various instructions differ slightly, usually regarding punctuation. If your format is not exactly what the journal uses, the copyeditor will make small changes. In the Editors’ opinion, this issue will not cause the rejection of a good paper.

## Literature review

The related literature should be reviewed, the most important published papers should be read in full text, and quality citations should be included in the references list [3]. References should be accurate and up-to-date. They should derive primarily from peer-reviewed journals, standard textbooks or monographs, or stable electronic sources. The authors generally should use the most relevant and novel studies from high-quality peer-reviewed sources. Importantly, electronically available citations should be preferred; abstracts and submitted articles (pending publication), newsletters, proceedings, and meetings syllabus should not be used because many in these categories ultimately do not pass peer review because it is not possible to be traced and cited [4–6].

While section editors and reviewers assigned as per their classifications will have some familiarity with the research area, often, they will not be as expert in the area as the authors are. Therefore, the authors should provide a clear, reader-friendly description focusing on critical issues in the research area. They should explain and convince the editors, the reviewers, and the readers that the study fills an essential gap in the literature, the idea is original, the rationale addresses conflicting reports, and possibly it might lead to a change in practice [4, 5]. This information should be given in the Introduction section of the manuscript.

## Authorship issues

The number and the order of the authors’ names should be fair by reflecting their contribution and the order of their contribution to the manuscript. All who authored should be listed as authors of the manuscript; none who did not author should be listed. Researchers who have contributed to the work, but not enough to merit their inclusion in the authorship, should be acknowledged in the respective section. For example, a scientist who contributed only to the statistical analysis but not to the study conception and design, data acquisition, drafting or critical revision of the manuscript could be acknowledged only [7]. Authorship is not a way to thank a colleague for support, access to resources, or mentorship [7]. Scientific misconduct (fraud) in authorship includes a gift or complimentary authorship, ghost authorship, and coercion authorship [3]. Any change in the number and order of the authorship after submission is generally not allowed, except if approved by the editor-in-chief [8].

Authors familiar with the journal, such as previous submission/publication and peer-reviewers, have an advantage. Indeed those authors who have frequently reviewed for the journal or have previously submitted a paper to be considered for publication at the journal usually have a far better vision of the scope and quality requirements of the journal in question. In this regard, this understanding will prove invaluable to prepare their next submission to the right quality level and increase the chances of it being accepted for publication.

## Abstract

After the title, the abstract is the first section of the manuscript, and for approximately 75% of the authors, it is the only section to read, either because of lack of time or lack of institutional access to the full text of the manuscript [9]. The abstract should also have an outline that should follow the journal’s instructions (structured or not). In any case, it should be a summary of the main sections; it should include the rationale/key questions of the study, should provide the materials, methods (basic procedures), and results, and should end with a discussion/conclusion that is relevant to the study question and comes out of the study methods and results. The editors, reviewers, and readers should be able to understand the study by just reading its abstract. Abbreviations should be avoided, and citations are not allowed in the abstract; in our opinion, *P*-values may also be omitted from the abstract.

Journals incorporate a word number limitation in the abstract. Therefore, the abstract should emphasize new and important aspects of the study. If the editor likes the abstract, they will most likely read the article itself [10–15].

## Topic

The topic of the study may be topical, which the editors do not like, but it may be of interest, which the editors do like. In general, the editors do not favor copycat topics, meaning topics that closely imitate, adopt, copy or mimic one another. The editors are not interested in yet another study of the same problem osteoporosis/hip fractures, femoral head osteonecrosis, volar plating for distal radial fractures, etc that replicate previous studies. In contrast, the importance of new research or a new way of looking at an old problem should be emphasized in scientific writing.

The topic of the study should be focused on; if it is too broad, it will produce an article much too long that is not easy to read and digest. Additionally, reporting authors’ own experience is not a reason for publication because it may not be relevant or of interest to other people unless important information, a new implant, a surgical technique, or modification of standard techniques/methods are presented.

## Type of study

An accurate level of evidence is required depending on the type of study. For randomized clinical trials, calculation of the sample size, a robust randomization method, and knowledge of the primary and secondary outcome measures and loss to follow-up are important. For matched cohort studies, the definition of the cohort and matching is important. The authors should clearly inform the readers if the study groups are comparable and any potentially confounding variables between the study groups at baseline. For registry studies, it is important to include where/what is the registry, and how complete and comprehensive the database is. Meta-analyses are overdone; for the meta-analysis to be interesting, it should include important study questions. If the answer to these questions is negative, then the meta-analysis is probably not necessary. If the answer to these questions is the need for high-level randomized trials, then the study is useless. In any study, concluding that there is a need for further research reduces the power of the study and discourages the further reading of the article.

## Type of writing

Medical writing should be formal and not confuse reading. Standard rules of grammar and syntax should be followed; redundancy should be avoided, and slang and idiomatic should not be used. Do not invent but use abbreviations that are widely known, such as ORIF (open reduction internal fixation), THA (total hip arthroplasty), or TKA (total knee arthroplasty). The editorial office can handle minor errors in writing and grammar; however, a poorly written article, full of typing errors and careless writing and preparation is more likely to be rejected.

Paragraphs should be indented and presented in an orderly, organized fashion according to concepts/ideas. Each paragraph (except for the paragraph of limitations and the final paragraph) should be supported by appropriate citations [4, 5]. Paragraphs should not be made too short or too long and should incorporate clear and short sentences related to a common topic. Long sentences and tortuous phrases become confusing and not understandable. Illustrations should show important features, be in the highest possible quality and be accompanied by explanatory legends; clinical photographs should be avoided because of patients’ identifier issues [9–17].

## Invited papers

Scientific journals such as SICOT-J and International Orthopaedics often ask once or twice a year for invited papers in special issues with one to three guest editors. The scope of these special issues is to focus on current, hot, and/or important topical topics. The invited papers aimed for these special issues are subjected to the same peer-review process by the journal’s reviewers, and if accepted, they are published in the same procedure [16, 17].

## Submission (cover) letter to the editor

A submission or cover letter to the editor should be the first item in the submission list. This should be addressed to the editor-in-chief and include information about the submitted paper and the corresponding author. A concise cover letter should tell the editor what is novel about the study and why it falls within the journal’s scope. For most journals, the submission letter should also include copyright and conflict of interest disclosures and author approval statements. A resubmission (cover) letter is also necessary for submitting revised manuscripts; this is often called the “Authors’ Responses to Reviewers’ comments” letter. These letters are important during the submission and review process as they provide important information for the original submission and the point-by-point answers and manuscript changes according to the editor’s and the reviewers’ comments and recommendations [6].

## Summary

Submitting a paper for publication means recognizing and respecting what the journal and the editors anticipate from the authors. What the editor likes from a submitted paper is a focused topic of interest, a well-written manuscript with an aim and scope that falls within the journal’s scope, a manuscript with a compact introduction/discussion section, clear study question, accurate statistics, appropriate illustrations, and tabular data. What the editor does not like from a paper is a careless outline, a copycat topic or a topic that does not fall within the aims and scope of the journal, a manuscript written in poor language, a long manuscript, an unclear study question, overstated conclusions, inaccurate statistics, too many figures and tables, and poor illustrations. The editors’ view is to maintain the journal’s reputation, raise the journal’s visibility, citation rate, and impact. However, this means a lot of work and stress for each submitted manuscript. Follow these tips to make it easier for the editor to accept your paper.

## Conflicts of interest

All authors declare no conflicts of interest.
